# Effect of enhanced plane of nutrition in early life on the transcriptome and proteome of the anterior pituitary gland in Angus heifer calves

**DOI:** 10.1186/s12864-024-10626-2

**Published:** 2024-08-02

**Authors:** Kate Keogh, Alan K. Kelly, David A. Kenny

**Affiliations:** 1 Teagasc Animal & Bioscience Research Department, Teagasc Grange, Dunsany, Co Meath Ireland; 2 School of Agriculture and Food Science, University College Dublin, Belfield, Dublin 4, Ireland

**Keywords:** Early life nutrition, Heifer, Puberty

## Abstract

**Background:**

Enhanced nutrition during the early calfhood period has been shown to lead to earlier pubertal development in heifer calves. This is of interest as earlier pubertal onset can subsequently facilitate earlier calving which can economically benefit production systems. Reproductive development in heifers is regulated by the hypothalamic-pituitary-ovarian signalling pathway. In particular the anterior pituitary gland is central to reproductive development, through the dynamics of gonadotropic pulsatility. However, despite clear knowledge of the influence of enhanced dietary intake on subsequent reproductive development, the molecular control governing this response in the pituitary gland within the hypothalamic-pituitary-ovarian signalling axis in heifer calves is not fully understood. The objective of this study was to examine the effect of an enhanced plane of nutrition during early life on the anterior pituitary gland of heifer calves through both transcriptomic and proteomic analyses. Between 3 and 21 weeks of age, heifer calves were offered either a high (HI, *n* = 14) or moderate (MOD, *n* = 14) plane of nutrition, designed to elicit target growth rates of 1.2 and 0.5 kg/d for HI and MOD groups, respectively. All calves were euthanised at 21 weeks of age and anterior pituitary tissue harvested for subsequent use in global transcriptomic and proteomic analyses.

**Results:**

Average daily gain was affected by diet (*P* < 0.001) and was 1.18 and 0.50 kg/day, for HI and MOD calves, respectively. RNAseq analysis resulted in the identification of 195 differentially expressed genes (P_adj_<0.05; fold change > 1.5), with 277 proteins identified as differentially abundant (P_adj_<0.05; fold change > 1.5) between contrasting dietary treatment groups. Biochemical pathway analysis of differentially affected genes and proteins revealed an enrichment for both growth hormone and GnRH signalling pathways (P_adj_.<0.05). Additionally, pathway analysis predicted an effect of enhanced dietary intake on endocrine function within the anterior pituitary gland as well as on reproductive system development and function (P_adj_.<0.05).

**Conclusions:**

Results from this study show that an enhanced dietary intake during early calfhood affected the molecular control of the anterior pituitary gland in heifer calves in early life.

**Supplementary Information:**

The online version contains supplementary material available at 10.1186/s12864-024-10626-2.

## Background

Early onset of puberty is a key trait underpinning economically efficient cattle production systems, due to the ability to facilitate calving at two years of age in heifers that reach puberty earlier in life [1]. In cattle, pubertal onset occurs in a gradual fashion, and the process is regulated by a range of biochemical processes, including interaction between metabolic, neuroendocrine and reproductive tissues [2–5]. Furthermore, the metabolic control towards reproductive development is not only apparent around the time of puberty onset. Research has shown that puberty onset can be hastened through early life dietary manipulation, specifically offering an enhanced diet to heifers during the early life period can subsequently lead to earlier puberty attainment, compared with heifers offered a typical early life growing diet [3, 5–7]. Indeed, studies have shown that improved nutritional status during early juvenile development has a much greater impact on advancing the maturation of the reproductive axis as well as pubertal age when compared to nutritional interventions at later developmental stages [8–11]. For example, Gasser et al. [9] showed that offering diets that promoted rapid weight gains between 3 and 7 months of age, resulted in heifers which reached puberty earlier when compared to a moderately fed group. Enhanced metabolic status during the early life period can be conveyed to the hypothalamus gland, which can then subsequently elicit down-stream physiological events, including reproductive development and sexual maturation, mediated by the hypothalamic-pituitary-ovarian (HPO) biochemical signalling axis in heifers [5, 8]. Indeed previous research from our own laboratory clearly shows that enhanced nutrition during early calfhood alters the biochemical regulation of the hypothalamus consistent with advanced sexual development in the prepubertal heifer [5]. Within the HPO axis, the anterior pituitary gland is crucial for the synthesis and subsequent secretion of hormones, following prior messaging from the hypothalamus gland. Moreover, the anterior pituitary regulates several physiological processes throughout the body including stress and growth as well as reproduction and lactation. Within the context of sexual maturation, and HPO signalling, GnRH produced within the hypothalamus signals to its receptor within the anterior pituitary gland, resulting in the synthesis and secretion of the gonadotropins, FSH and LH. In females, FSH and LH in turn signal to their receptors on the ovary resulting in gonadal development and the secretion of estrogen, which is responsible for pubertal attainment [12, 13].

However, although the anterior pituitary is known as a crucial mediator towards reproductive development and subsequent puberty attainment, knowledge of the underlying molecular control regulating this effect remains to be fully elucidated. A greater understanding of the underlying biology governing the relationship between enhanced metabolic status and earlier reproductive development would allow for the potential identification of molecular biomarkers that could be used in the context of genomic selection breeding programs to breed cattle better able to reproductively respond to enhanced metabolic status in early life. Moreover, improved knowledge of this effect could also contribute to the development of optimal calf rearing management systems for the earlier attainment of puberty in heifers. Thus, given the aforementioned known influence of enhanced metabolic status during the early life period on reproductive development and subsequent earlier pubertal onset, the objective of this study was to evaluate the effect of an enhanced dietary intake during the early-life period on the molecular response of the anterior pituitary tissue through transcriptomic and proteomic analyses in heifer calves compared to contemporaries fed a typical moderate plane of nutrition.

## Results

### Animal performance

The effect of differential feeding on growth performance is outlined in full in Kelly et al. [8]. Daily energy intake over the entire trial consisting of both milk replacer and concentrate was 2.5 times higher for the HI group (*n* = 14) compared to the MOD group (*n* = 14; *P* < 0.001) [8]. Specifically for this, calves were offered milk-feeding plans during the pre-weaning phase consisting of initial allowances of 10 and 4 L of milk replacer for days 0–30 for HI and MOD groups, respectively. For both groups, these allowances were gradually reduced to 0 L of milk replacer by day 56 of the experiment. During the post-weaning phase, HI calves were offered concentrate *ad libitum*, whilst MOD calves were offered 1 kg of concentrate per day. Differential feeding resulted in differences in growth rates between the two groups. These diets, as fully described in Kelly et al. [8] were designed to reflect a typical standard growing diet (MOD) and a diet designed to allow animals to reach their full metabolic requirements (HI). Over the entire experimental trial, HI calves grew at 1.18 kg/d, whilst MOD calves grew at 0.5 kg/d (*P* < 0.001), resulting in HI calves being on average 76.6 kg heavier than MOD calves (*P* < 0.001) at the time of tissue collection at 21 weeks of age,

The effect of differential feeding in early life on physiological parameters and indices of reproductive development of the heifers used in this study are described in full in Kelly et al. [8]. Briefly, systemic concentrations of insulin, glucose and IGF-1 were greater in HI calves compared to MOD calves, whilst systemic concentrations of FSH were greater in MOD compared to HI (*P* < 0.001). Additionally, over the duration of a GnRH challenge FSH and estradiole concentrations were lower and higher for HI and MOD groups, respectively (*P* < 0.05). The weight of the total reproductive tract, uterus and ovarian tissue relative to bodyweight were all greater for HI calves compared to MOD calves (*P* < 0.05), whilst ovarian surface follicle numbers were also greater (*P* < 0.05) in the HI calves.

### mRNAseq analyses

An average of 46 million sequencing reads were generated through mRNA sequencing across all anterior pituitary samples (HI: *n* = 14; MOD: *n* = 14). Alignment of trimmed sequencing reads to the bovine genome resulted in an average mapping rate of 89.6% across all samples. Following the removal of lowly expressed genes within edgeR, a total of 14,002 genes remained for differential expression analysis. EdgeR analysis resulted in the identification of 195 DEGs (P_adj_ <0.05; fold change > 1.5) between treatment groups. Differentially expressed genes and their fold changes are presented in Additional Table S1. Raw sequencing reads and gene counts for each sample utilised in this study have been deposited within NCBI’s Gene Expression Omnibus and are available through GEO ID GSE230543.

### Global proteomics analysis

Protein quantification and identification of anterior pituitary samples (HI: *n* = 12; MOD: *n* = 12), undertaken through MaxQuant software (v1.6.2.3) resulted in the identification of 4,813 proteins across the pituitary samples examined. The total number of proteins represents those with at least two peptides and a maximum of 10 missing values per protein. Of the 4,813 proteins identified within the anterior pituitary, 277 were identified as significantly differentially abundant (P_adj_.<0.05; fold change > 1.5) between dietary treatment groups. The full list of proteins identified in the anterior pituitary tissue are presented in Additional Table S2. Proteomics data generated in this analysis have been uploaded to the ProteomeXchange Consortium via the PRIDE (http://www.ebi.ac.uk/pride) partner repository [14] with the data identifier PXD042011.

### Pathway analysis of transcriptome and proteome datasets

**Fig. 1:**
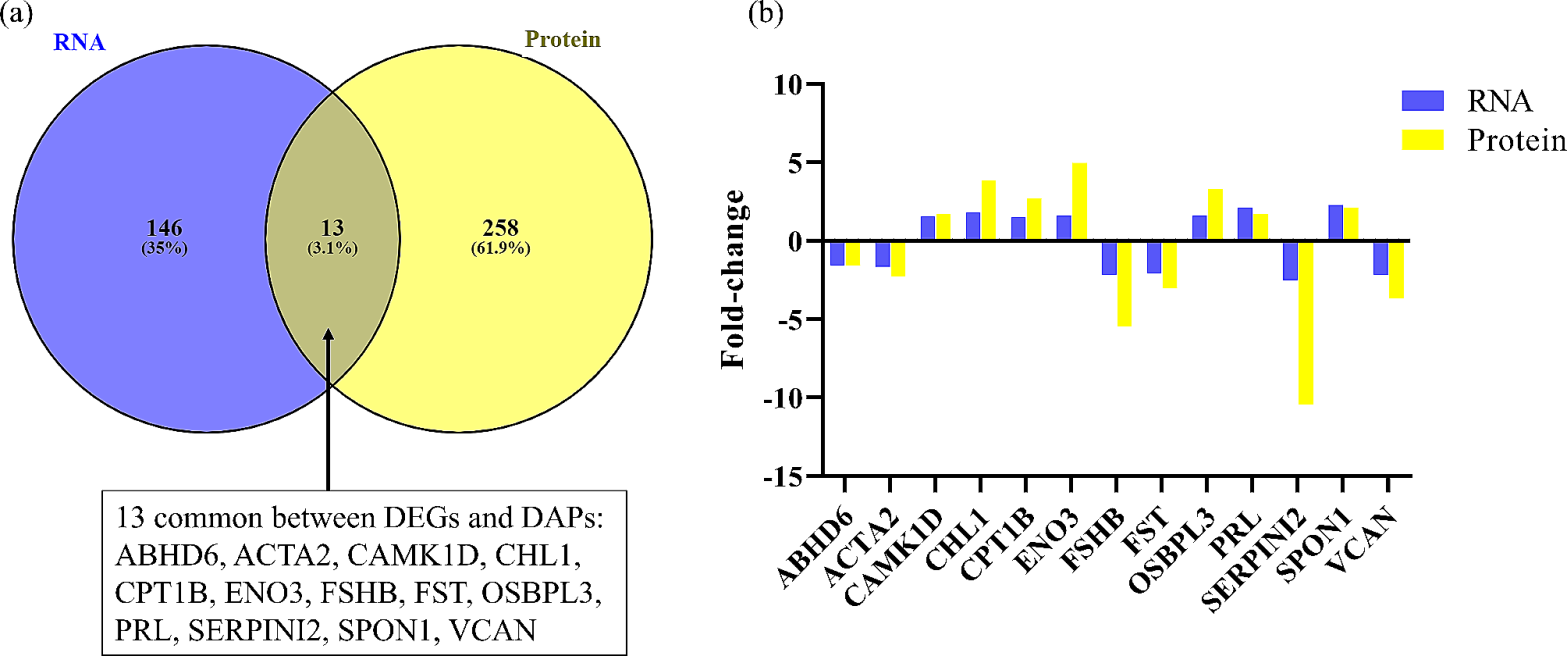
Comparison between differentially expressed genes and differentially abundant proteins. (**a**) Venn diagram showing 13 molecules as commonly affected by enhanced early life nutrition in the anterior pituitary gland across transcriptome and proteome datasets; (**b**) comparison of direction of effect of 13 molecules commonly identified as differentially expressed and differentially abundant in transcriptome and proteomic datasets, respectively. Fold changes are positive or negative in the HI calves relative to the MOD calves

**Fig. 2:**
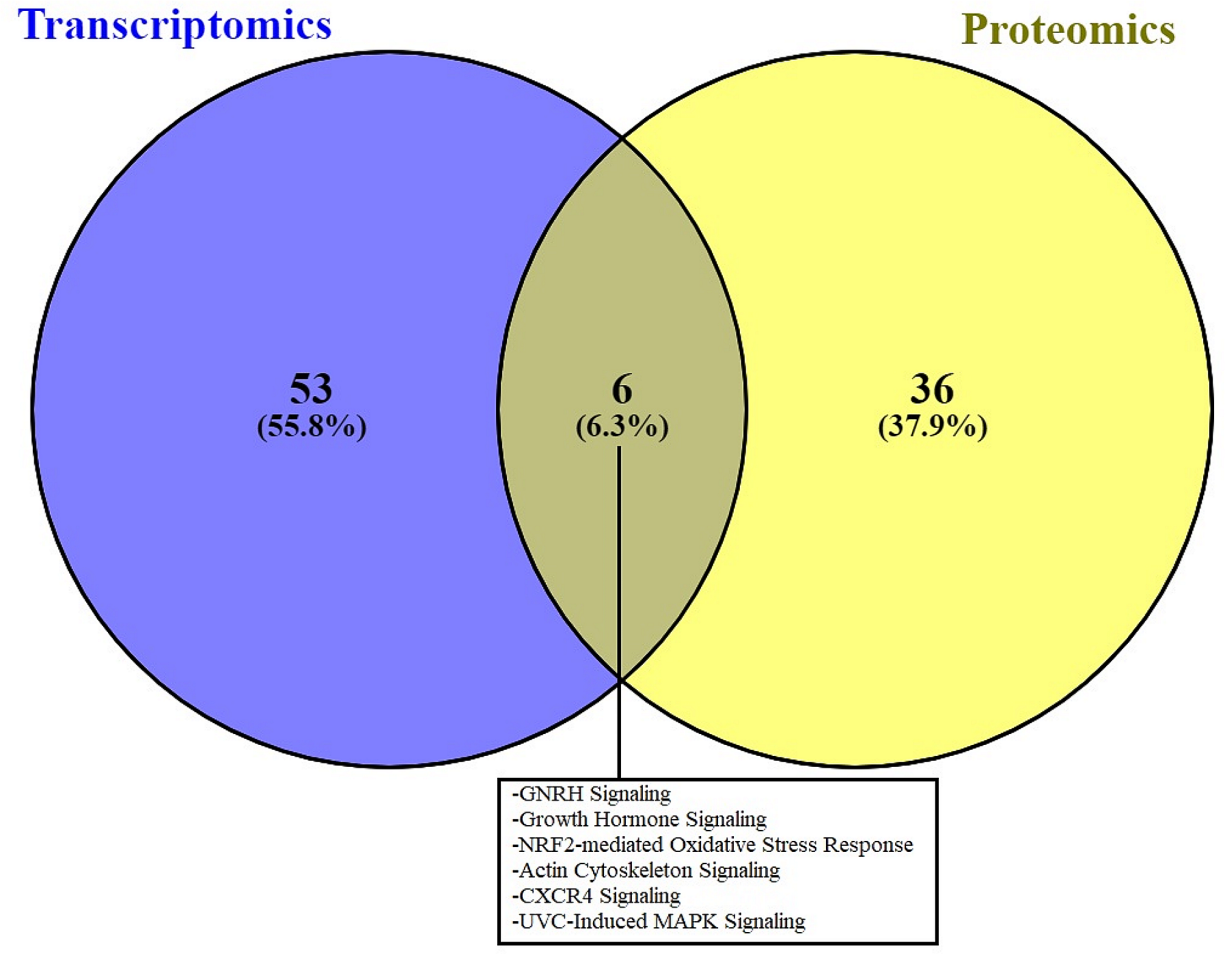
Comparison between biological pathways significantly enriched between HI and MOD calves for both transcriptomic and proteomic analyses

**Fig. 3:**
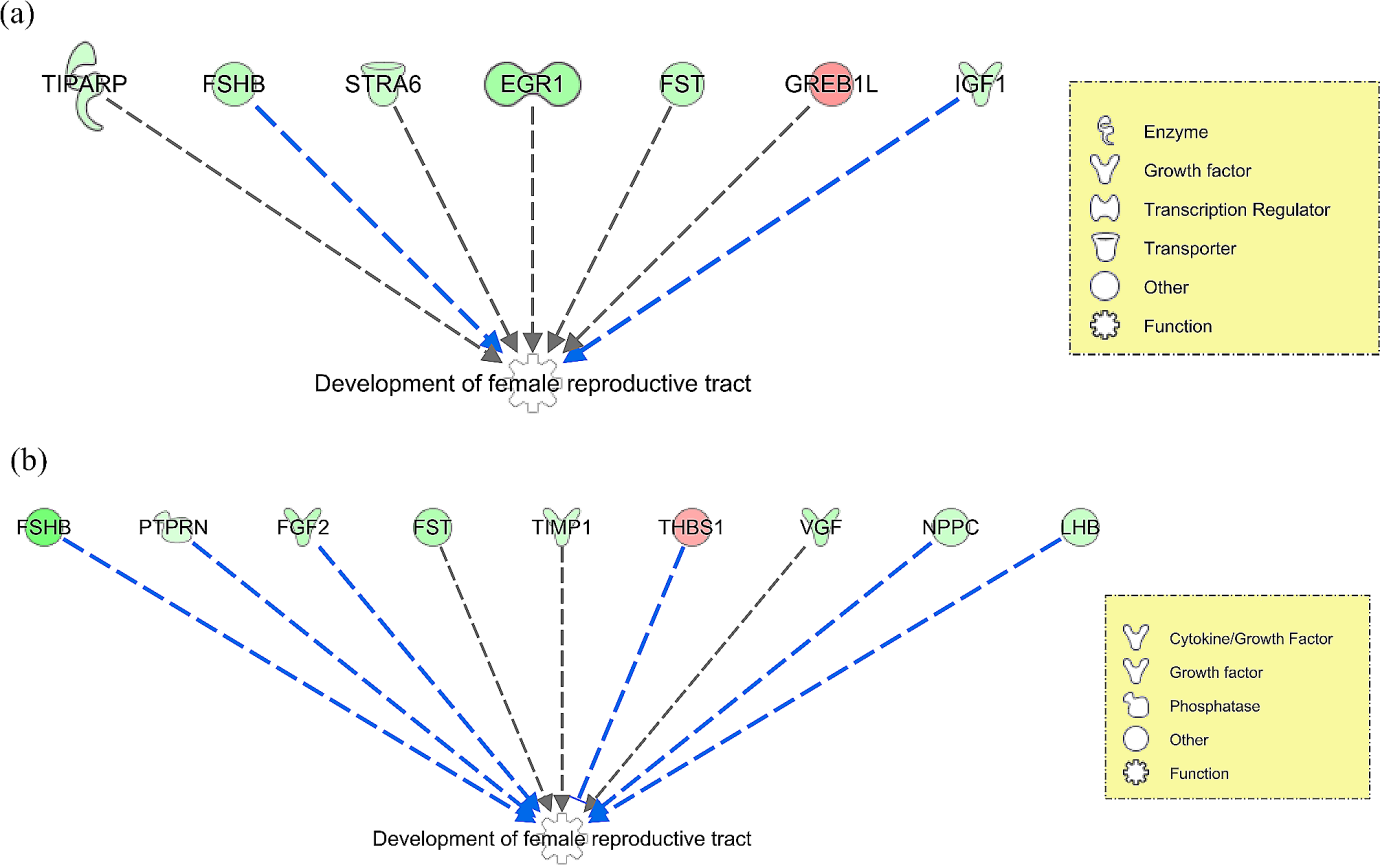
Development of female reproductive tract biological process affected by enhanced dietary intake in the (**a**) transcriptome and (**b**) proteome of the anterior pituitary of heifer calves. Lines between molecules describe the relationship as predicted in IPA [44]; blue line: leads to inhibition; black line: effect not predicted

**Fig. 4:**
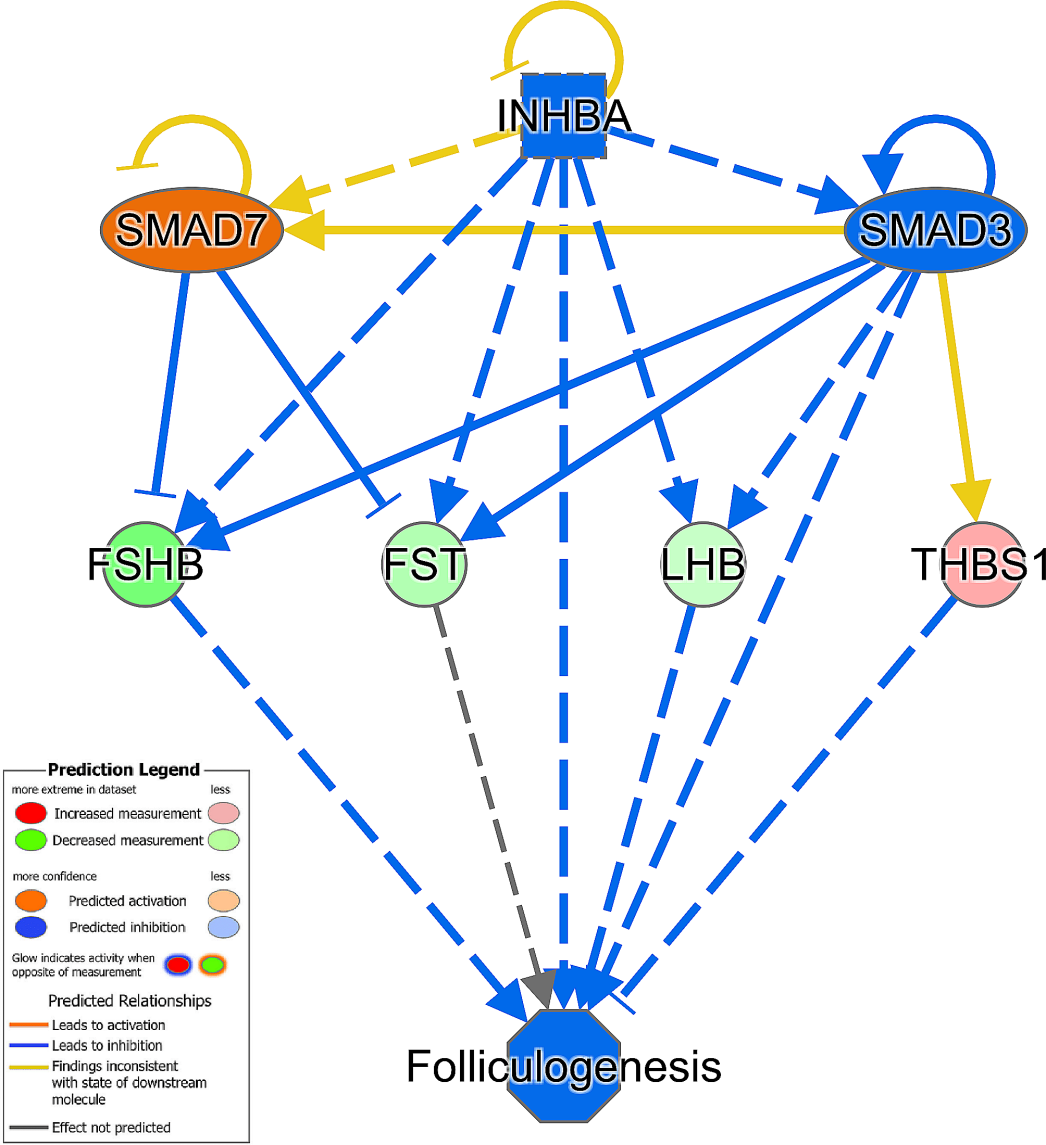
Differentially abundant proteins (green or red molecules) identified as affecting the biological process of folliculogenesis through IPA analysis [44] in heifer calves as a consequence of varied plane of nutrition in early life

Within IPA, 159 DEGs and 271 DAPs were successfully mapped to the IPA knowledge base for biochemical and biological pathway analysis. A comparison between the DEGs and DAPs revealed only 13 genes and proteins commonly differentially affected by early life plane of nutrition in the anterior pituitary (Fig. 1). Indeed, an evaluation of DEGs based on the exact animals used for proteomics revealed the commonality of only one additional gene, the actin-binding protein *TAGLN* which was down-regulated in the HI calves across both RNAseq and proteomics analyses when the same animals were used for each analysis. However, despite a relatively small number of common genes and proteins, pathway analysis revealed enrichment of the same biological processes in each transcriptomic and proteomic datasets (Fig. 2). For example, canonical pathway analysis revealed enrichment of GnRH and growth hormone signalling pathways as well as actin cytoskeleton and MAPK signalling in the anterior pituitary between HI and MOD fed calves (Additional tables S3 and S4 for transcriptomic and proteomic datasets, respectively). Additionally biological processes related to the development of the female reproductive tract and endocrine system development were also significantly enriched between treatment groups across both transcriptomic and proteomic datasets. Differentially expressed genes and DAPs related to the development of the female reproductive tract as determined by IPA are shown in Fig. 3 (a) and (b) respectively. Additionally, also through IPA folliculogenesis was predicted to be affected based on DAPs, proteins identified as different between treatment groups and involved in folliculogenesis are shown in Fig. 4.

## Discussion

The current study aimed to investigate the underlying molecular control in the pituitary gland in response to enhanced nutritional intake in heifer calves up to 21 weeks of age utilising global transcriptomics and proteomics analyses. Anterior pituitary samples were harvested at 21 weeks of age in order to capture a period during which enhanced dietary intake is known to affect reproductive development and subsequent pubertal onset [8–11]. However, despite identifying large numbers of transcripts and proteins different between the dietary treatment groups, a comparison of commonality between DEGs and DAPs revealed only a small subset of molecules as commonly affected by early life dietary intake across each analysis undertaken. This outcome was potentially not unexpected given previous reports from both our own group [15, 16] and others [17] where the agreement between global transcriptome and proteome data has been reported to be relatively low. Notwithstanding the potentially low number of genes and proteins identified as different across both datasets, the direction of effect was the same for proteins/genes commonly affected across both analyses, providing confidence in the results. However, despite this outcome, commonality across the two datasets was apparent for the enrichment of biological pathways and processes as a direct response to early life differential feeding strategies. For example, pathway analysis revealed enrichment of both GnRH and growth hormone signalling pathways, as well as biological processes related to female reproductive tract development and endocrine functions related to pituitary development and hormone processing. Thus, the remainder of this discussion will focus on these biological pathways and functions enriched within both transcriptome and proteome analyses.

### Growth hormone signalling

Growth hormone is an anabolic hormone and together with IGF forms the somatotropic axis [18, 19], thus it is not surprising that varying plane of nutrition and consequent differences in growth rate during the first 21 weeks of life resulted in enrichment of the growth hormone signalling pathway across both transcriptome and proteome analyses. However, whilst it might have been expected for the growth hormone signalling pathway to be up-regulated in the HI group due to their greater dietary intake and consequent greater propensity for growth, genes differentially expressed within the growth hormone signalling pathway suggests less of a role for somatotropic signalling towards pituitary development as a consequence of enhanced early life nutrition. Specifically DEGs including *GHRHR*, the receptor for growth hormone releasing hormone; the anabolic hormone *IGF1* and *CSHL1*, which encodes a somatotropin involved in the regulation of growth were all down-regulated in the HI group. However, other genes and proteins enriched within the growth hormone signalling pathway were up regulated in the HI calves. These included the genes *PRKCB* and *PRL* and the DAPs; PRKCA, PRL and SLC2A4. PRKCA and *PRKCB* are members of the protein kinase C (PKC) family of serine-threonine protein kinases. Members of the PKC family of intracellular second messengers phosphorylate a wide variety of protein targets and are known to be involved in diverse signalling pathways, affecting many different cellular processes such as cell adhesion, cell cycle regulation and cellular proliferation [20]. Interestingly, *PRKCB* was identified within a gene co-expression network of the arcuate nucleus of the hypothalamus of the same heifer calves used in the current study [5]. In that study *PRKCB* was co-expressed in a network also containing the *NEBL* gene, which was previously implicated towards puberty through both differential gene expression between pre- and post-puberty stages [21] and SNPs associated with puberty development [22], suggesting a potential role for *PRKCB* towards earlier reproductive development as a consequence of enhanced dietary intake in early life. The anterior pituitary hormone, prolactin, encoded by the *PRL* gene functions as a growth regulator for many tissues. Similar to the current study *PRL* was also up-regulated in the hypothalamic tissue of the heifer calves used in this study [5]. The greater expression and abundance of SLC2A4, which is responsible for the insulin-regulated transport of glucose in the HI calves is also in agreement with the documented greater systemic insulin concentrations of the HI calves [8]. Moreover, pathway analysis highlighted insulin as an upstream regulator of *PRL* and *PRKCB* expression and PRL, PRKCA and SLC2A4 abundance in transcriptome and proteome datasets, respectively, suggesting a role for insulin towards development of the pituitary gland as well as greater insulin sensitivity in the pituitary gland compared to the MOD groups of calves. Overall, the down-regulation of genes of the somatotropic axis as well as the up-regulation of transcripts and proteins involved in insulin response suggests a role for insulin, ahead of IGF/GH towards development of the anterior pituitary following 18 weeks of enhanced dietary intake in heifer calves.

### Endocrine processes

One of the primary functions of the pituitary gland is to synthesise and then secrete several hormones important to overall body function, including those related to growth, metabolism and reproduction [23]. Indeed, in line with differing metabolic status between HI and MOD calves used in the current study [8] differences in genes and proteins related to endocrine functions within the anterior pituitary were apparent. This was manifested through an enrichment of processes related to endocrine system development and function. Specifically these included the following processes enriched from DEGs: metabolism, synthesis, concentration and secretion of hormones as well as enrichment for processes related to peptide hormone processing and catabolism of hormones based on DAPs. Differentially expressed genes involved in hormone processing including *CACNA1E*, *DIO3*, *CASR*, *ETV1*, *DRD2* and *ECE1* were all up-regulated in the HI calves, indicating a greater degree of hormonal processing within the anterior pituitary as a consequence of greater dietary intake in early life. Evidence for an effect of enhanced early life nutrition on the thyroid gland was also established through the differential expression of *CASR* and *DIO3* which are involved in thyroid hormone regulation. The thyroid hormones are required for the normal functioning of nearly all tissues with major effects on oxygen consumption and metabolic rate, thus contributing to overall animal maintenance energy requirements [24]. Belonging to the iodothryonine deiodinase family, *DIO3* catalyses the inactivation of thyroid hormone, thereby regulating the thyroid hormones and playing a crucial role in mammalian development [25]. Indeed the thyroid hormone receptor, THRA was identified as a regulator for the expression of *DIO3* within the transcriptome dataset. The *CASR* gene encodes a plasma membrane G-protein coupled receptor, sensitive to changes in circulating calcium concentration, coupling cellular calcium concentration to intracellular signalling pathways capable of modifying parathyroid hormone secretion [26]. This gene also displayed greater expression in the anterior pituitary following enhanced dietary intake in a contemporary study undertaken in bull calves up to 18 weeks of age [27]. Similar to *CASR*, the *CACNA1E* gene is also responsive to concentrations of cellular calcium. *CACNA1E* encodes a voltage-dependent calcium channel, involved in calcium dependent hormone and neurotransmitter release and was also up-regulated in the HI calves. Conversely, the same gene was down-regulated in the HI calves in the hypothalamic arcuate nucleus [5]. Differential expression of *ETV1* was of particular interest due to its role in enhancing POMC transcription within the pituitary gland [28]. Pro-opiomelanocortin (POMC) neurons form an integral part of the central melanocortin system regulating feeding behaviour, specifically functioning in appetite suppression [29]. Up-regulation of *ETV1* may also contribute to greater expression of *POMC*, however although such a result was not apparent within the current study in both datasets, differential expression of genes within the arcuate nucleus of the same calves used in the current study revealed greater expression of *POMC* in the HI calves [5]. This was in agreement with down-regulation of appetite stimulant genes in the same tissue including *NPY* and *AGRP* [5], overall indicating a lower appetite in the HI calves following 18 weeks of an enhanced dietary regimen. Finally, the greater expression of *DRD2*, which encodes a dopamine receptor in HI calves coincided with the greater expression of *PRL*, demonstrating the role of *DRD2* in prolactin regulation [30]. Moreover *DRD2* has also been shown to be involved in the regulation of other hormones involved in growth, food intake and glucose metabolism. For example Garcia-Tornadu et al. [30] reported an effect of growth hormone levels and increased dietary intake in mice depleted of the *DRD2* gene implicating a role for this gene towards dietary intake and growth processes within the anterior pituitary. Overall results indicate a greater propensity for hormonal processing in the HI calves concomitant with their greater dietary intake and subsequent metabolic processing and growth requirements.

In addition to the aforementioned effect of varied plane on nutrition on the hormones IGF1 and PRL, differential expression and abundance of the gonadotropins, FSHB and LHB, was also apparent within the anterior pituitary. FSHB was down-regulated in the HI calves in both transcriptome and proteome datasets, whilst LHB was down-regulated in the proteomic data. Enhanced early life nutrition is known to hasten puberty attainment, mediated through earlier secretion of the gonadotropins, FSH and LH, however results from the current study indicate lower transcript abundance of gonadotropin concentrations in the calves on the HI plane of nutrition. Indeed, this was also apparent through lower systemic concentrations of both FSH and LH in the HI calves used for the current study [8]. Kelly et al. [8] also reported greater systemic concentrations of estradiole in the HI calves, determining that the lower concentrations of the gonadotropins was due to a gradual decline in the sensitivity of the hypothalamic/anterior pituitary axis to the negative feedback of estradiol [31], as a consequence of the acceleration in the onset of increased LH pulse frequency due to enhanced dietary intake. However, despite the greater systemic concentrations of estradiol in the HI calves, lower expression of *ABHD6*, whose expression is regulated by estrogen, in HI calves indicated towards a lower estrogen concentration within the HI calves. However the lack of differential expression of estrogen receptors in either the anterior pituitary transcriptome or proteome dataset suggest that this may be due to the receptors targeting the hypothalamus as opposed to the anterior pituitary gland. Overall results indicate that the processing of the gonadotropins within the anterior pituitary was altered as a consequence of an enhanced plane of nutrition up to 21 weeks of age in these heifer calves.

### GnRH signalling

In addition to the effect of plane of nutrition on the two gonadotropins, FSHB and LHB, other molecules within the GnRH signalling pathway were also affected. Binding of GnRH to its receptors triggers a complex array of intracellular signal transduction events within the gonadotrophs; these signalling cascades orchestrate the overall physiological response of these cells to GnRH stimulation, culminating in the synthesis and release of the gonadotropins, LH and FSH [32]. In addition to differential expression and abundance of FSHB and LHB, genes of the GnRH signalling pathway differentially expressed included *CACNA1E*, *CACNA1I*, *EGR1*, *GNA15*, *PRKCB* and *RASD1*, whilst the following proteins displayed differential abundance between treatment groups: MAP2K1, MAPK10, PAK4 and PRKCA. Some of these genes/proteins (*CACNA1E*, *PRKCB* and PRKCA) have been discussed above in relation to growth hormone signalling, due to the two pathways sharing similar intracellular signalling and signal transduction cascades upon growth hormone and GnRh receptor binding. Similar to *CACNA1E*, *CACNA1I* encodes a subunit of a voltage gated calcium channel, responding to cellular levels of calcium and eliciting subsequent responses. Both *CACNA1E* and *CACNA1I* can contribute to hormone or neurotransmitter release and both were up-regulated in the HI calves. Moreover, *GNA15* was also up-regulated in HI calves in the transcriptome dataset, with this gene involved in G-protein couple receptor binding activity but in response to calcium ion concentration. Within the GnRH signalling pathway, both *CACNA1E* and *CACNA1I* contribute to calcium influx which subsequently leads to activation of PKC [33], indeed the *PRKCB* and *PRKCA* subunits of PKC were also up-regulated in the HI calves as discussed previously. Additionally, MAPK10, a member of the MAP family of kinases as well as PAK4, an activator of MAP kinases both displayed greater abundance within the proteomic data of the HI calves. As part of the GnRH signalling pathway members of the MAPK intracellular signallers contribute to the gene expression of the gonadotropins [34], however equally through their role as intracellular signalling molecules, these kinases contribute to various other cellular signalling pathways. Within the current study, both FSHB and LHB displayed lower expression and abundance in the HI calves, suggesting potential inhibition of gonadotropin synthesis in the HI calves following 18 weeks of enhanced dietary intake. Conversely though another member of the MAPK family, MAP2K1, as well as a member of the Ras superfamily of small GTPases (RASD1) both displayed lower abundance in the HI calves. However, Haisenleder et al. [35] highlighted the use of a MAP2K1 inhibitor resulted in the prevention of GnRH-induced increases in gonadotropin mRNA. Indeed the estrogen receptor pathway includes MAP2K1, suggesting a potential method for estrogen to elicit negative feedback and prevent greater expression of gonadotropins within the HI calves. Additionally, down-regulation of the *EGR1* gene provides further evidence for an inhibition of gonadotropin synthesis within the HI calves, due to its function in regulating the biosynthesis of LHB within the pituitary gland [36]. Moreover, *EGR1* was also down-regulated in the hypothalamic arcuate nucleus of the HI calves used in the current study [5]. Overall, the differential expression and abundance of genes and proteins of the GnRH signalling pathway indicate towards an up-regulation of molecules involved in gonadotropin gene expression, but ultimately down-regulation of the gonadotropins, consistent with negative feedback by estradiol as suggested previously by the data of Kelly et al. [8].

In addition to the enrichment of the GnRH signalling pathway, GnRH was also identified as an upstream regulator within the proteomic dataset. GnRH was identified as an upstream regulator controlling the abundance of FSHB, LHB, CHGA and SCG2. The latter two proteins, CHGA and SCG2 are of particular interest due to the identification of SNPs within these genes previously associated with puberty attainment in heifers [21, 22]. The CHGA protein is a member of the chromogranin/secretogranin family of neuroendocrine secretory proteins, acting as a negative regulator of the neuroendocrine system [37]. Whilst the SCG2 protein is also a member of the chromogranin/secretogranin family, functioning in the packaging and sorting of peptide hormones and neuropeptides into secretory vesicles [37]. Furthermore, two additional members of the secretogranin family, SCGN and SCG5 both also displayed lower protein abundance within the HI calves used in the current study. Similarly *SCGN* expression was also lower in the hypothalamus of the calves used in the current study, but conversely *CHGA* expression was greater in the arcuate nucleus of the HI calves [5]. In addition, a recent gene co-expression analysis conducted on pituitary tissue of bull calves fed either a HI or low diet up to 18 weeks of life reported networks of co-expressed genes containing CHGA and secretogranin genes as significantly associated with concentrations of both metabolic (IGF1) and reproductive (LH, testosterone) hormones [38]. Altogether, results from the current study as well as others within the published literature highlight the potential importance of the secretogranin genes to reproductive development as a consequence of enhanced early life dietary intake and thus warrant further investigation.

### Development of the female reproductive tract

Due to the aforementioned differential expression and abundance of genes and proteins involved in GnRH signalling as well as hormone processing, the development of the female reproductive tract was identified as an affected biological process within IPA as a consequence of varied plane of nutrition in early life in both transcriptome and proteome datasets. Proteins and genes contributing to this biological effect are presented in Fig. 3 and from this figure it can be seen that the majority of the DEGs and DAPs were down-regulated in the HI calves. These included *EGR1*,* FSHB*,* FST*,* IGF1*,* STRA6* and *TIPARP* in the transcriptome dataset and FGF2, FSHB, LHB, NPPC, PTPRN, TIMP1 and VGF in the proteomic dataset. Although some of these genes have been discussed above in relation to negative feedback of estradiol, additional genes and proteins highlighted as affecting the development of the female reproductive also appear to be dependent on estradiol regulation. For example, lower expression of *STRA6* has been associated with greater concentrations of estradiol in endometritis [39]; NPPC has been shown to contribute to oocyte meiosis in granulosa cells, and is also responsive to E2 concentrations [40]; and FGF2 has been shown to be involved in estrogen regulation of pituitary lactotroph mitogenesis [41]. However, although genes and proteins with apparent functions dependent on estrogen levels were different between HI and MOD dietary groups, differential expression or abundance of estrogen receptor was not apparent within the current study. This is further established through the down-regulation of *TIPARP* in the transcriptome dataset, which functions in the regulation of estrogen receptor transcription. Despite no effect of early life dietary intake on estrogen receptor genes within the pituitary, the *GREB1L* gene was up-regulated in the HI calves. The *GREB1L* gene encodes the GREB1 like retinoic acid receptor coactivator, and although this gene has been shown to have a role in development or reproductive organs [42], its primary gene, GREB1, encodes an estrogen receptor which may mediate estrogen action [43]. Thus, there is potential for the *GREB1L* gene to also function as an estrogen receptor, moreover, the greater expression of this gene within the HI calves would suggest a function for this gene as an estrogen receptor, which may be contributing to the negative feedback of estrogen towards gonadotropin synthesis within the anterior pituitary gland.

Other genes and proteins contributing to reproductive system development included VGF, PTPRN, FST and THBS1. VGF is of particular interest due to its primary role in the regulation of energy homeostasis [44]. Hypothalamic VGF expression has been shown to be regulated by both feed restriction and leptin, with VGF knockout mice displaying increased adiposity, reduced energy expenditure and impaired glucose tolerance. Additionally there is evidence that VGF peptides may also be involved in the control of reproduction since VGF-knockout mice displayed delayed puberty, reduced fertility and decreased ovarian and uterine weights [44]. Moreover LH and FSH subunits at pituitary levels were reduced in VGF-null mice [44]. Although this gene displayed lower expression in the HI calves, evidence of delayed or perturbed reproductive tract development was not apparent in the calves used in the current study [8], evident through greater numbers of ovarian surface follicles and oocytes recovered as well as component weights of the female reproductive tract reported in the heifer calves offered the high energy diet during the first 21 weeks of life. Similarly, *FST* and *PTPRN* also displayed lower expression in the HI calves. *PTPRN* encodes a member of the protein tyrosine phosphatase family of signalling molecules and has been shown to be required for normal accumulation and secretion of pituitary hormones including FSH and LH. Whilst FST encodes follistatin which functions in the inhibition of FSH release, with lower expression of this gene potentially a direct response to lower expression of FSHB within the pituitary. Similarly the lower abundance of LHB may have contributed to the greater expression of *THBS1*, whose expression is inhibited by LH signals [45]. The differential expression of FST, THBS1 as well as the gonadotropins (FSHB, LHB) also contributed to an effect on folliculogenesis, which is the process by which the female germ cell develops within the somatic cells of the ovary and matures into a fertilizable egg. This effect was predicted within pathway analysis to be regulated by members of the TGF-beta superfamily of proteins, including INHBA, SMAD3 and SMAD7. INHBA functions within the pituitary gland to inhibit the secretion of FSH, however differential expression or abundance of INHBA was not apparent within the current study. Overall results from this study clearly highlight an effect of varied dietary intake in early life on the development of the female reproductive tract in both transcriptome and proteome datasets.

## Conclusions

Results from this study clearly show that dietary augmentation in early life affects both the transcriptome and proteome of the anterior pituitary of heifer calves. Despite a relatively low level of agreement between genes differentially expressed and proteins identified as differentially abundant between the HI and MOD dietary treatment groups, biological processes were commonly enriched across both transcriptome and proteomic datasets. These included enrichment of pathways including growth hormone signalling and GnRH signalling as well as enrichment of biological processes related to endocrine system development and function and development of the female reproductive tract. Results from this study provide evidence to support a greater understanding of the underlying biology governing the relationship between enhanced metabolic status and earlier reproductive development which could also potentially contribute to earlier puberty attainment and will allow for the potential identification of molecular biomarkers, following appropriate validation, that could be used in the context of genomic selection breeding programs to breed cattle better able to reproductively respond to enhanced metabolic status in early life.

### Methods

All procedures involving animals were approved by the Teagasc Animal Ethics Committee and were performed in accordance with the relevant guidelines and regulations including the ARRIVE guidelines (Animal Research: Reporting of In Vivo Experiments). This study was licensed by the Health Products Regulatory Authority (licence number AE19132/P061) in accordance with the European Union Directive 2010/36/EU.

### Animal model

Tissue samples used in this study were derived from a larger study aimed at uncovering the impact of plane of nutrition during early calfhood on the physiological and molecular control of sexual development in the heifer calf [8], the background experimental design is only briefly described here. All calves used in this study were sourced and purchased from commercial farms in Ireland. Twenty-eight Angus × Holstein-Friesian heifer calves with a mean (± SD) age and bodyweight of 19 (± 4) days and of 51.2 (± 7.8) kg, respectively, were blocked on age, bodyweight, farm of origin and genetic merit and allocated within block to one of two dietary plane of nutrition groups: High (HI, *n* = 14) or Moderate (MOD, *n* = 14). These diets, as fully described in Kelly et al. [8] were designed to reflect a typical standard growing diet (MOD) and a diet designed to allow animals to reach their full metabolic requirements (HI). Daily dietary allowances were formulated to support target average growth rates of > 1.2 kg/day and 0.50 kg/day for the HI and MOD nutritional treatments, respectively, until the calves reached 21 weeks of age. All calves were individually offered milk replacer and concentrate from the beginning of the trial up until weaning using an electronic feeding system. Calves within the HI group were offered a milk feeding plan as follows: Stage I (days 0–30), 10 L of reconstituted milk replacer; Stage II (days 30–35), 10 L of reconstituted milk replacer gradually reduced to 6 L; Stage III (days 35–42), 6 L of reconstituted milk replacer and; Stage IV (days 42–56), 6 L of reconstituted milk replacer gradually reduced to 0 L. Moderately-fed calves (MOD) were offered a milk feeding plan as follows: Stage 1 (days 0–50) 4 L of reconstituted milk replacer; Stage II (days 50–56), 4 L of reconstituted milk replacer gradually reduced to 0 L. Milk replacer (20% fat and 26% protein) was reconstituted to 15.0% solids. Additionally, HI calves were offered concentrate *ad libitum*, whilst MOD calves received a stepped-up allowance, peaking at a maximum of 1 kg of concentrate per day during the week of weaning. Hay was also provided as a source of roughage (250 g/hd/day) and all calves also had *ad libitum* access to water. During the post-weaning phase of the trial, HI calves were offered concentrate *ad libitum*, whilst MOD calves were offered 1 kg of concentrate per day. Both treatment groups were offered hay to appetite during the post-weaning phase. Throughout the trial, all calves were weighed regularly on a weekly basis. At 21 weeks of age (145 ± 3 days), all calves were euthanized.

### Tissue isolation

Anterior pituitary samples were harvested as previously described in English et al. [27]. At a mean age of 21 weeks (145 ± 3 days), all calves were euthanized using an intravenous overdose of phenobarbital. Following euthanisation and confirmation of death, the brain was removed from the skullcap by severing the infundibulum, optic nerves and brain stem. The pituitary gland was then removed from the sella turcica and both anterior and posterior sections of the pituitary gland were separated. All instruments used for tissue collection were sterilised and treated with RNAzap prior to use. All pituitary tissue samples were washed in Dulbecco’s phosphate-buffered saline (DPBS), and subsequently snap frozen in liquid nitrogen. Tissue samples were subsequently stored at -80° C pending further processing.

### Transcriptomic analysis

#### RNA isolation and RNA sequencing

Methodology used for RNA isolation and subsequent sequencing has been described in full in Keogh et al. [46] and is only briefly outlined here. The Qiagen RNeasy Plus Universal kit (Qiagen, UK) was used to isolate RNA from all anterior pituitary samples, according to the manufacturers instructions. RNA samples were then quantified and quality assessed using a Nanodrop spectrometer and Agilent Bioanalyzer, respectively, ensuring all samples were of high quality (RNA integrity number (RIN) > 8). cDNA libraries were subsequently prepared from 1 µg of total RNA for each sample using the Illumina Truseq stranded mRNA kit (Illumina, San Diego, CA, USA). Sequencing of cDNA libraries was then undertaken on an Illumina Novaseq platform employing 150 bp paired-end sequencing. Bioinformatic analysis was undertaken as per Keogh et al. [46]. Briefly, sequencing reads were first checked for quality using FastQC (version 0.11.7 [47]), following which sequencing adapters were removed using cutadapt software (version 1.18.8 [48]). Reads were then aligned to the bovine reference genome (ARS-UCD-1.2 [49]) using STAR (version 2.5.2.b [50]), whilst also employing the quantmode function in order to quantify the number of sequencing reads aligned to each gene. EdgeR (version 3.20.9 [51]), was then used to determine genes differentially expressed between the two contrasting dietary treatment groups. Genes with a Benjamini-Hochberg false discovery rate of 5% and a fold-change greater than 1.5 were considered differentially expressed. Pathway analysis of DEGs was undertaken using Ingenuity pathway analysis (Qiagen; [52]) in order to assign biological annotation and undertake biological pathway analysis.

### Global proteomics analysis

#### Sample preparation

Global proteomics analysis was undertaken on the same cohort of samples used for RNAseq (HI, *n* = 12; MOD, *n* = 12). Details related to sample processing and liquid chromatography-mass spectrometry analysis are described in full in Coen et al. [15]. Briefly, proteins were extracted from each sample using a tissue homogenizer (TissueLyser II, QIAGEN). The solubilization of the extracted proteins was then enhanced by processing the samples with High Intensity Focused Ultrasound (HIFU) for 1 min setting the ultrasonic amplitude to 85%. Protein concentration was determined using the Qubit^®^ Protein Assay Kit (Life Technologies, Zurich, Switzerland). Following protein extraction, the samples were digested using the iST Kit (PreOmics, Germany).

#### Liquid chromatography-mass spectrometry analysis

Following protein extraction and subsequent digestion mass spectrometry analysis was performed on a Q Exactive HF-X mass spectrometer (Thermo Scientific). The acquired raw mass spectrometry data were processed using MaxQuant (version 1.6.2.3 [53]), followed by protein identification using the integrated Andromeda search engine. Spectra were searched against a Uniprot Bos Taurus reference proteome [49]. The maximum false discovery rate (FDR) was set to 0.01 for peptides and 0.05 for proteins. Protein fold changes were computed based on intensity values. A set of functions implemented in the R package SRMService [53] was used to filter for proteins with 2 or more peptides allowing for a maximum of 10 missing values, and to normalize the data with a modified robust z-score transformation and to compute p-values using the t-test with pooled variance. Significantly differentially abundant proteins (DAP; P_adj_ <0.05; fold change > 1.5) were subsequently subjected to biochemical pathway analysis in IPA (Qiagen; [52]) in order to assign biological annotation and undertake biological pathway analysis.

### Electronic supplementary material

Below is the link to the electronic supplementary material.

Supplementary Material 1

## Data Availability

The transcriptomic dataset generated for this study can be found in the NCBI’s Gene Expression Omnibus (GEO) database [https://www.ncbi.nlm.nih.gov/geo/] (GEO accession ID: GSE230543; https://www.ncbi.nlm.nih.gov/geo/query/acc.cgi?acc=GSE230543). The proteomic dataset generated for this study can be found in the ProteomeXchange Consortium via the PRIDE (http://www.ebi.ac.uk/pride) partner repository with the data identifier PXD042011 (https://www.ebi.ac.uk/pride/archive/projects/PXD042011).

## Abbreviations

DAP: Differentially Abundant Protein
DEG: Differentially Expressed Gene
DPBS: Dulbecco’s Phosphate Buffered Saline
FDR: False Discovery Rate
FSH: Follicle Stimulating Hormone
GEO: Gene Expression Omnibus
GnRH: Gonadotropin Releasing Hormone
HI: High Plane Of Nutrition Treatment Group
HIFU: High Intensity Focused Ultrasound
HPO: Hypothalamic-Pituitary-Ovarian
IPA: Ingenuity Pathway Analysis
LH: Luteinizing Hormone
MOD: Moderate Plane Of Nutrition Treatment Group
PCR: Polymerase Chain Reaction
RIN: RNA Integrity Number
SD: Standard Deviation
